# Wearable respiratory sensors for COVID‐19 monitoring

**DOI:** 10.1002/VIW.20220024

**Published:** 2022-10-31

**Authors:** Guorui Chen, Sophia Shen, Trinny Tat, Xun Zhao, Yihao Zhou, Yunsheng Fang, Jun Chen

**Affiliations:** ^1^ Department of Bioengineering University of California, Los Angeles Los Angeles California 90095 USA

**Keywords:** biomarkers, COVID‐19, point‐of‐care, respiratory sensors

## Abstract

Since its outbreak in 2019, COVID‐19 becomes a pandemic, severely burdening the public healthcare systems and causing an economic burden. Thus, societies around the world are prioritizing a return to normal. However, fighting the recession could rekindle the pandemic owing to the lightning‐fast transmission rate of SARS‐CoV‐2. Furthermore, many of those who are infected remain asymptomatic for several days, leading to the increased possibility of unintended transmission of the virus. Thus, developing rigorous and universal testing technologies to continuously detect COVID‐19 for entire populations remains a critical challenge that needs to be overcome. Wearable respiratory sensors can monitor biomechanical signals such as the abnormities in respiratory rate and cough frequency caused by COVID‐19, as well as biochemical signals such as viral biomarkers from exhaled breaths. The point‐of‐care system enabled by advanced respiratory sensors is expected to promote better control of the pandemic by providing an accessible, continuous, widespread, noninvasive, and reliable solution for COVID‐19 diagnosis, monitoring, and management.

## Graphical abstract

Wearable respiratory sensors can monitor biomechanical signals such as the abnormities in respiratory rate and cough frequency caused by COVID‐19, as well as biochemical signals such as virus biomarkers from exhaled breaths. The point‐of‐care system enabled by advanced wearable respiratory sensors is expected to promote better control of the pandemic by providing an accessible, continuous, widespread, noninvasive, and reliable solution for COVID‐19 diagnosis, monitoring, and management.

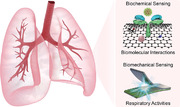

## 1 INTRODUCTION

Coronavirus disease 2019 (COVID‐19), caused by severe acute respiratory syndrome coronavirus 2 (SARS‐CoV‐2), has spread across the globe and developed into a pandemic since its outbreak at the end of 2019, with more than 600 million infections and over 6.3 million deaths.^1^, ^2^, ^3^ Infected people suffer from abundant health problems in the heart (for example, acute myocarditis, arrhythmias, heart failure), lungs (for example, dyspnea, lung inflammation, and fibrosis), nervous system (for example, loss of the sense of taste and smell), digestive system (for example, gastrointestinal disturbance with diarrhea), and musculoskeletal system (for example, extreme fatigue, muscle ache).^4^, ^5^, ^6^ These severe health threats have shrunk the world economy with lower growth, limited production, and supply chain disruptions.^7^ In the United States,^8^ gross domestic product fell 32.9% in the second quarter of 2022, which was the deepest decline since 1947. To return to normal in the context of COVID‐19, society is prioritizing restarting the economies safely while avoiding the resurgence of the virus in their post‐pandemic policies.^9^, ^10^, ^11^ However, SARS‐CoV‐2 possesses a lightning‐fast transmission rate with a basic reproductive number (R_0_) of 2.2, able to infect others even before symptom onset.^12^ With recovering social economic activities and international commerce, effective and widespread testing of COVID‐19 is essential to interrupt infection transmission and allow for the implementation of timely treatment.^13^

Currently, methods for identifying infected individuals rely on detecting viral genetic material from a nasal or throat swab.^14^, ^15^, ^16^ However, molecular tests possess many disadvantages. First, continuous and long‐term monitoring of COVID‐19 is required since a negative test result only means that the individual was not infected at the time of sample collection or the incubation period was not over.^17^ During the incubation period, the individuals get tested negative but can still infect others. Meanwhile, it is possible that immediately after the test sample was taken, the individual became infected with the virus. Secondly, highly accurate molecular tests are not widely available.^18^ They rely on limited single‐use kits. Thus, frequent and universal molecular tests for population‐level that are extremely reliable will not be feasible. Moreover, as daily life begins to resume to normal, the only method of COVID‐19 detection is through random sampling or self‐screening. However, this lacks the ability to implement large‐scale and accurate monitoring of the virus and contains a high risk for exposure. Finally, the molecular test samples must be analyzed at centralized medical facilities.^19^ The transportation of samples is inconvenient and requires trained staff, increasing time delay and impeding the prompt diagnosis of COVID‐19, which can lead to infection by asymptomatic individuals. Therefore, to ease the burden of COVID‐19 on global public health and return to normal daily activities,^20^ there is an urgent need for accessible, continuous, widespread, noninvasive, and reliable technologies to diagnose and monitor COVID‐19 infections.

**FIGURE 1:**
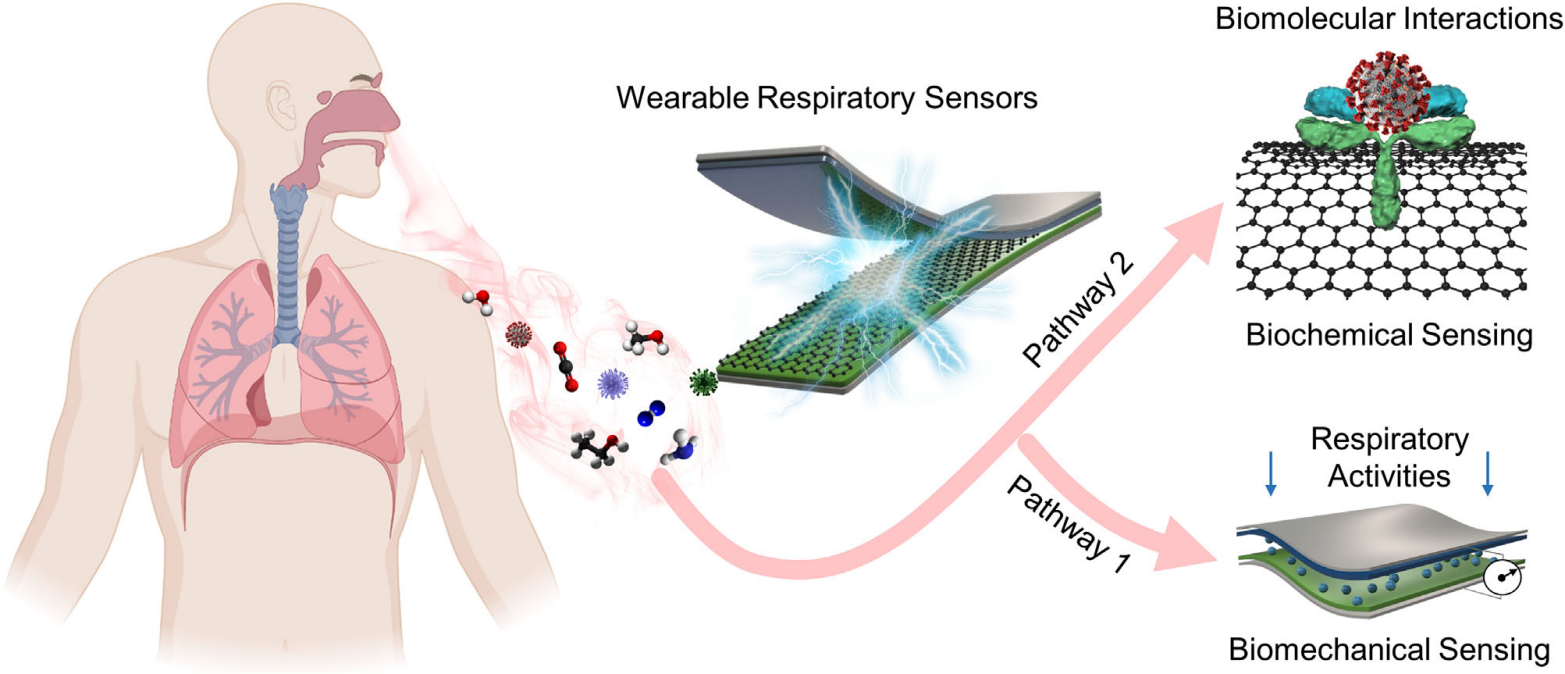
Pathways of wearable respiratory sensors for COVID‐19 monitoring. Leverage wearable respiratory sensors for biomechanical monitoring, such as the respiratory rate and cough frequency (pathway 1), as well as biochemical monitoring, such as exhaled breath analysis (pathway 2), which will provide an accessible, continuous, widespread, noninvasive, and reliable solution toward COVID‐19 diagnosis, monitoring, and management. Partially created with BioRender.com

Abnormalities in respiratory activities, such as dry cough, difficulty in breathing, and shortness of breath, have been clinically proven to be one of the earliest signs of COVID‐19 infection.^21^, ^22^, ^23^ Meanwhile, viral agents induce specific volatile organic compounds (VOCs), which can reach the exhaled breaths, thereby working as a fast diagnostic biomarker for COVID‐19.^24^ Thus, direct measurement of respiratory biomarkers, for example, respiratory rate, cough frequency, cough intensity, respiratory effort, and on‐site viral‐specific VOCs,^25^ represent a potential game‐changer in the diagnosis, monitoring, and management of COVID‐19.^26^ To date, bioelectronics, the convergence of biological systems and electronic devices, have witnessed tremendous progress in wide applications,^27^, ^28^, ^29^, ^30^, ^31^, ^32^, ^33^ such as biosensing,^34^, ^35^, ^36^, ^37^, ^38^, ^39^, ^40^, ^41^, ^42^, ^43^, ^44^ electrical stimulations,^45^, ^46^, ^47^ neuromodulation,^48^ drug delivery,^49^, ^50^, ^51^ energy harvesting,^52^, ^53^, ^54^ energy storage,^55^, ^56^, ^57^ thermoregulation,^58^ display,^55^ imaging,^59^ and many others. Specifically, respiratory sensors propelled by advanced bioelectronic technology have been widely used for health assessment, disease diagnosis, and treatment tracking in daily life.^60^, ^61^, ^62^ Now, respiratory sensors have the potential of transforming toward point‐of‐care systems to cope with the COVID‐19 outbreak by detecting subtle abnormalities in respiratory activities as well as analyzing exhaled breaths.^63^, ^64^, ^65^ In this Mini Review, we summarize current technological advances in respiratory sensors and their implementations for point‐of‐care monitoring of COVID‐19. As illustrated in Figure 1, we highlight the respiratory sensors for biomechanical signal monitoring, such as the respiratory rate and cough frequency (pathway 1), as well as biochemical signal monitoring,^66^ such as exhaled breath analysis (pathway 2).^67^ Wearable point‐of‐care system enabled by advanced respiratory sensors is expected to promote better control of the pandemic via diagnosis by providing accessible, continuous, widespread, noninvasive, and reliable monitoring of COVID‐19.

## 2 BIOMECHANICAL SENSING OF RESPIRATORY ACTIVITIES

**FIGURE 2:**
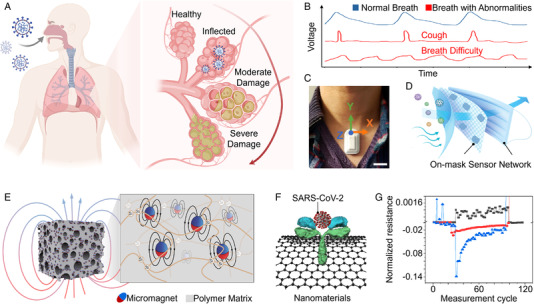
Biomechanical sensing of respiratory activities and biochemical sensing of exhaled breath for COVID‐19 monitoring. COVID‐19 can cause lung complications and usually lead to respiratory problems. Created with BioRender.com. (B) Continuous respiratory signals monitoring: normal breath pattern (a fairly steady rate), cough pattern (voltage peaks with enhanced amplitude in a short time), as well as breath difficulty pattern (voltage signals with low amplitude and high frequency). (C) A suprasternal notch‐mounted sensing patch for respiratory monitoring. Scale bar, 2 cm. Reproduced under the terms of the Creative Commons Attribution License 4.0 (CC BY).^66^ Copyright 2021, National Academy of Sciences. (D) An on‐mask sensor network for adaptive respiratory monitoring. Reproduced with permission.^73^ Copyright 2022, Wiley. (E) The giant magnetoelastic effect in a soft material system. Reproduced with permission.^77^ Copyright 2021, Springer Nature. (F) Exhaled breath analysis for SARS‐CoV‐2 detection. (G) Representative electrical resistance response of the hybrid sensor array to three different breath samples. Black, an infected patient. Red, a cured patient. Blue, a healthy control. Reproduced under the terms of the Creative Commons Attribution License 4.0 (CC BY).^67^ Copyright 2020, American Chemical Society

COVID‐19 can cause lung complications and usually lead to respiratory problems such as dry cough, difficulty breathing, and shortness of breath (Figure 2A).^68^, ^69^, ^70^ Thus, leveraging respiratory sensors for tracking abnormalities in respiratory activities can effectively detect potential COVID‐19 cases and monitor their respiratory health status. Compared with normal respiratory activities, which have a fairly steady rate, those infected generate a rapid and repetitive cough with forceful airflows (Figure 2B). Moreover, those with COVID‐19 will have more cumulative coughs as the disease progresses and decrease as they recover. A clinical study has demonstrated that one COVID‐19 patient saw a decreasing trend in cumulative coughs as they recovered over an 8‐day period.^71^ By integrating known patterns such as this with machine learning, both diagnosis and recovery states for COVID‐19 patients can be accurately determined. Meanwhile, the breathing difficulty that also accompanies COVID‐19 is marked by rapid but shallow breaths, which can also be detected through a measure of respiratory rate. These acquired signals can then be wirelessly transmitted to personal terminals such as a smartphone for further clinical‐level analysis.

To detect variations in respiratory activities, respiratory sensors can be worn as a chest strap,^72^ integrated on a mask,^73^ or mounted on the suprasternal notch in a patch form.^66^ In this manner, biomechanical signals induced by respiratory activities, such as the movement of the chest, air flow of inhaled/exhaled breaths, and suprasternal retractions, can be continuously tracked with high fidelity. Until now, respiratory sensors have widely been developed based on typical mechanisms of human body‐powered triboelectric,^74^ piezoelectric,^75^, ^76^ and magnetoelastic,^77^, ^78^, ^79^ and nonself‐powered capacitive,^80^ and resistive effects.^81^ Commonly used materials for biomechanical sensing include polytetrafluoroethylene, polydimethylsiloxane, polyimide, poly(vinylidene fluoride), metallic nanomaterials, MXenes, liquid metals, graphene, carbon nanotubes, etc.^82^, ^83^, ^84^ For example, a suprasternal notch‐mounted sensing patch supported by a thin, double‐sided biomedical adhesive was developed to determine coughing, respiratory rate, heart rate, speech patterns, and physical activity (Figure 2C). Then, using the acquired data, the device was able to utilize a convolutional neural network to determine any abnormalities in the acquired data, and whether those abnormalities are indicative of symptoms associated with COVID‐19.^66^ These results demonstrate that distinctive features in respiratory biomarkers between COVID‐19 patients and healthy subjects can be used for long‐term disease diagnosis, monitoring, and management.

Besides these respiratory sensors, which require external power, human body‐powered devices have also been extensively investigated for respiratory activities monitoring. For example, triboelectric nanogenerators (TENGs) can effectively convert ubiquitous biomechanical signals into electric signals and have sparked a human body‐powered sensor revolution in the field of point‐of‐care. Recently, an on‐mask sensor network consisting of multiple textile TENGs was developed for adaptive respiratory monitoring (Figure 2D).^73^ When triboelectric sensing nodes are subjected to exhaled airflow, the electron cloud of two atoms from different triboelectric material surfaces will strongly overlap, lowering the potential barrier and allowing for electron transition, thereby, generating a current in the external circuits. The generated signals contain personalized information on respiratory intensity and respiratory frequency. By using deep learning to analyze these biomechanical signals, the on‐mask sensor network can realize respiratory pattern recognition with high classification accuracy. These results demonstrate that this system can provide reliable rehabilitation monitoring and assistance for those COVID‐19 patients.

Very recently, a textile magnetoelastic generator (MEG) based on the giant magnetoelastic effect in a soft material system (Figure 2E)^77^ was developed as a fundamentally new approach for respiratory monitoring.^79^ The MEG was based on the recently discovered magnetoelastic effect in soft polymer systems in Chen Group at the University of California, Los Angeles.^77^ A typical MEG consists of two components: a magnetomechanical coupling (MC) layer and a magnetic induction (MI) layer.^85^, ^86^, ^87^ By stitching the textile MEG around the chest area of a nursing scrub, the expansion and contraction of the ribcage induced by respiratory activities deform the MC layer, and shift its magnetic field, thus generating a current output in the MI layer. The current signals reliably recorded the frequency, intensity, and persistency of different respiratory patterns such as normal breathing, rapid breathing, and coughing. Assisted by machine learning algorithms, these respiratory patterns can be distinguished with cross‐validation mean accuracy of up to 90.89%, demonstrating the potential for COVID‐19 monitoring. More importantly, the MEG is intrinsically waterproof without encapsulation, which is suitable for respiration monitoring with a high‐mean sweating rate or exhalation rate with much moisture.

In brief, biomechanical signals related to respiratory activities measured by such respiratory sensors are highly important for rapid diagnosis of COVID‐19 and can be a valuable supplement to molecular tests to allow for continuous, accessible, widespread, noninvasive, and reliable monitoring of COVID‐19. In the future, more research efforts directed toward extracting various respiratory parameters from the biomechanical sensor‐collected signals, such as respiratory effort and lung capacity, are desired.^88^ Meanwhile, to prove the reliability of the wearable respiratory sensors, the derived parameters should be validated with results from clinical methods.^89^ In addition to directly measuring the airflow or thoracic motions, other methods can be utilized for detecting respiratory abnormalities of COVID‐19, such as measuring respiratory sounds by detecting oronasal, thoracic, and tracheal signals.

## 3 BIOCHEMICAL SENSING OF EXHALED BREATH

In addition to the abnormities in respiratory activities, the airway and lung infections caused by SARS‐CoV‐2 can also lead to a variation of microbial flora,^25^ thereby conceivably resulting in the release of characteristic VOCs via the exhaled breaths.^90^ Pilot studies have revealed that some VOCs, such as methylpent‐2‐enal, 2,4‐octadiene 1‐chloroheptane, and nonanal,^25^ are correlated with viral infection with typical concentrations of 10–250 ppb.^24^, ^91^ Thus, biochemical sensing of exhaled breaths is expected to be an accessible, noninvasive, real‐time point‐of‐care method for large‐scale screening and monitoring of COVID‐19.^92^, ^93^, ^94^ Moreover, around a third of infectious people with COVID‐19 remain asymptomatic,^95^ causing solely monitoring of respiratory activities to cease effectiveness and raise potential risks for public health. Developing respiratory sensors for exhaled breath analysis will aid in the detection of SARS‐CoV‐2 and promptly rule out any suspected COVID‐19 cases in earlier stages (Figure 2F).^96^, ^97^, ^98^ Until now, many electrochemical biosensors have been developed for viral biomarker detection by leveraging various nanomaterials,^99^ such as conducting polymer (for example, poly(3,4‐ethylenedioxythiophene),^100^ metal‐based nanoparticles (for example, Au, Ag),^101^ and carbon materials (for example, carbon nanotubes, graphene sheets). ^102^ For example, a hybrid sensor array consisting of different gold nanoparticles linked to organic ligands was developed to detect COVID‐19 in exhaled breaths.^67^ When exposed to VOCs associated with COVID‐19, the sensing layer can interact with these biomarkers with a volume change (swelling/shrinkage), leading to a detectable increase/decrease of electric resistance (Figure 2G). To test, a pilot study was conducted with 140 participants, including 49 confirmed COVID‐19 patients, 58 healthy controls, and 33 non‐COVID lung infection controls. Then, using machine learning methods, the pattern of the output signals could be analyzed for the presence of COVID‐19, having a 76% accuracy and 100% sensitivity in differentiating those with and without COVID‐19.

To further enhance the wearability of biochemical sensing devices, an intelligent face mask was developed to detect the coronavirus spike protein and whole virus aerosol.^100^ This point‐of‐care system consists of a sub‐100 nm nanowire array‐based immunosensor for targeted viral particle capture, a miniaturized circuit for impedance measurement, and a Bluetooth module for result transmission. This system successfully demonstrated viral detection with a low concentration of 7 pfu/ml in only 5 min, convincing its potential for COVID‐19 screening as well as disease spread mitigation.

Although these results validate that a chemoresistance sensor can be used for point‐of‐care detection of COVID‐19, to date, respiratory sensors still have been extremely challenging to directly detect SARS‐CoV‐2 from exhaled breath owing to the very low viral load. Meanwhile, the concentration of the biomarkers is also easily influenced by the breathing protocol and the ambient environment. Innovations from multidiscipline researchers are highly required to accelerate the development of biochemical sensing to detect virus‐specific biomarkers from exhaled breaths, ultimately providing a reliable point‐of‐care screening solution and lowering the burden on hospitalization.

## 4 OUTLOOKS

The demand for global economic recovery motivates innovative technologies for continuously monitoring COVID‐19 to suppress viral spread. Point‐of‐care respiratory sensors can provide an accessible, continuous, widespread, noninvasive, and reliable solution for monitoring COVID‐19 in daily routines, allowing clinical services to shift from centralized medical facilities to everyday life. With these proven advantages, point‐of‐care respiratory sensors can be a promising tool to mitigate the spread of infectious respiratory diseases, effectively lowering the burden on the public healthcare system in an economical way. To promote this, ongoing research should focus on the following aspects.

### 4.1 Sensing performance

To perform continuous monitoring of COVID‐19, wearable respiratory sensors need to demonstrate long‐term stability under the erosion of sweat, body and ambient temperature fluctuations, mechanical deformations, and many other environmental impacts. Thus, precisely encapsulating respiratory sensors could be a critical research direction. Meanwhile, many respiratory sensors can respond to abundant biomechanical motions such as swallowing, speaking, heartbeat, and limb movements, which may mask respiratory information and introduce noise. Developing a differential respiratory monitoring system by deploying multiple sensing nodes in different locations of the human body is a possible solution to cancel artifacts. In addition, for biochemical sensing, developing nanomaterials with optimized selectivity on SARS‐CoV‐2‐related proteins, for example, spikes, envelope, matrix, or nucleocapsid proteins, could allow for ultrasensitive and low‐noise detection of biomarkers from exhaled breaths.

### 4.2 System integration

The respiration process contains various biomarkers for disease diagnosis, monitoring, and management. However, current respiratory sensors are standalone biomechanical or biochemical sensing, limited to a single biomarker detection. Developing respiratory sensors for simultaneous biomechanical and biochemical monitoring will provide a comprehensive analysis of respiration,^103^ ultimately promoting the management of COVID‐19. Meanwhile, point‐of‐care respiratory sensing systems with embedded data process circuits and wireless transmission modules will allow for a personalized analysis anywhere in any time frame, benefiting the mitigation of diseases in highly distributed populations.

### 4.3 Clinical data analysis

After collecting the sensing signals by using respiratory sensors, artificial intelligence and machine learning algorithms in the cloud could extract features associated with COVID‐19 from these signal datasets, providing streams of data for physicians to diagnose the disease even before the patient is conscious of it. However, current medical information infrastructures are complex and might interface the leakages of personalized health information. Therefore, much work is desired to develop an ethical and privacy‐ensured data sharing protocol and strengthen the safety of the wearable respiratory system. Meanwhile, regulatory procedures should be proposed to monitor the collection, release, share, and utilization of clinical data. Another challenge is the difference in various respiratory sensors and the individual health variations, which might impede the applicability of wearable respiratory sensors in a large and broader population. A worldwide calibration and evaluation standard is highly desired to unify the collecting, normalizing, and storing procedures of wearable respiratory sensing data.

In summary, point‐of‐care respiratory sensors hold great potential to continuously monitor COVID‐19 on a population level, providing enhanced benefits compared to current molecular tests such as ease of access and continuity. Moreover, for confirmed cases, point‐of‐care respiratory sensors can follow up on their respiratory health status, allowing for a rigorous screening solution at home or reflecting treatment progress at the hospital. Thus, point‐of‐care respiratory sensors will offer both physicians and the general public a continuous, economically viable, convenient, and customizable pandemic control tool in the fight against COVID‐19. We anticipate that the thoughts and perspectives in this article can arouse a wide range of research interests in developing point‐of‐care respiratory sensors for low‐cost and noninvasive detection of COVID‐19, emerging with multidiscipline research, facilitating economic recovery, and addressing the ongoing pandemic in our society.

## CONFLICT OF INTEREST

The authors declare no conflict of interest.

## FUNDING INFORMATION

Henry Samueli School of Engineering and Applied Science and the Department of Bioengineering, the University of California; Hellman Fellows Research Grant; UCLA Pandemic Resources Program Research Award; UCLA Academic Senate

## References

[1] D. K. Chu , E. A. Akl , S. Duda , K. Solo , S. Yaacoub , H. J. Schünemann , D. K. Chu , E. A. Akl , A. El‐harakeh , A. Bognanni , T. Lotfi , M. Loeb , A. Hajizadeh , A. Bak , A. Izcovich , C. A. Cuello‐Garcia , C. Chen , D. J. Harris , E. Borowiack , F. Chamseddine , F. Schünemann , G. P. Morgano , G. E. U. Muti Schünemann , G. Chen , H. Zhao , I. Neumann , J. Chan , J. Khabsa , L. Hneiny , L. Harrison , M. Smith , N. Rizk , P. Giorgi Rossi , P. AbiHanna , R. El‐khoury , R. Stalteri , T. Baldeh , T. Piggott , Y. Zhang , Z. Saad , A. Khamis , M. Reinap , S. Duda , K. Solo , S. Yaacoub , H. J. Schünemann , Lancet 2020, 395, 1973.32497510

[2] J. Bedford , D. Enria , J. Giesecke , D. L. Heymann , C. Ihekweazu , G. Kobinger , H. C. Lane , Z. Memish , M.‐d. Oh , A. A. Sall , A. Schuchat , K. Ungchusak , L. H. Wieler , Lancet 2020, 395, 1015. 32197103 10.1016/S0140-6736(20)30673-5 PMC7270596

[3] A. Telenti , A. Arvin , L. Corey , D. Corti , M. S. Diamond , A. García‐Sastre , R. F. Garry , E. C. Holmes , P. S. Pang , H. W. Virgin , Nature 2021, 596, 495. 34237771 10.1038/s41586-021-03792-w

[4] Z. Al‐Aly , Y. Xie , B. Bowe , Nature 2021, 594, 259. 33887749 10.1038/s41586-021-03553-9

[5] E. A. Troyer , J. N. Kohn , S. Hong , Brain. Behav. Immun. 2020, 87, 34. 32298803 10.1016/j.bbi.2020.04.027 PMC7152874

[6] Q. Xiong , M. Xu , J. Li , Y. Liu , J. Zhang , Y. Xu , W. Dong , Clin. Microbiol. Infect. 2021, 27, 89. 32979574 10.1016/j.cmi.2020.09.023 PMC7510771

[7] M. J. Dennis , Enroll. Manag. Rep. 2020, 24, 3.

[8] D. Dong , G. Gozgor , Z. Lu , C. Yan , Appl. Econ. 2021, 53, 1311.

[9] M. B. Allen , M. Mirsaeidi , Front. Public Health. 2020, 8, 235. 32574304 10.3389/fpubh.2020.00235 PMC7266873

[10] J. B. Bump , F. Baum , M. Sakornsin , R. Yates , K. Hofman , BMJ 2021, 372, n73. 33483317 10.1136/bmj.n73 PMC7819150

[11] T. L. Herron , T. Manuel , Bus. Soc. Rev. 2022, 127, 343.

[12] G. Chowell , K. Mizumoto , Lancet 22020, 395, 1093. 32247381 10.1016/S0140-6736(20)30743-1 PMC7270048

[13] S. K. Paul , P. Chowdhury , M. A. Moktadir , K. H. Lau , J. Bus. Res. 2021, 136, 316. 34538979 10.1016/j.jbusres.2021.07.056 PMC8437773

[14] G. Pascarella , A. Strumia , C. Piliego , F. Bruno , R. Del Buono , F. Costa , S. Scarlata , F. E. Agrò , J. Intern. Med. 2020, 288, 192. 32348588 10.1111/joim.13091 PMC7267177

[15] J. Beauté , C. Adlhoch , N. Bundle , A. Melidou , G. Spiteri , Lancet Infect. Dis. 2021, 21, 1344. 34450053 10.1016/S1473-3099(21)00461-8 PMC8384351

[16] O. Vandenberg , D. Martiny , O. Rochas , A. van Belkum , Z. Kozlakidis , Nat. Rev. Microbiol. 2021, 19, 171. 33057203 10.1038/s41579-020-00461-z PMC7556561

[17] J. Watson , P. F. Whiting , J. E. Brush , BMJ 2020, 369, m1808. 32398230 10.1136/bmj.m1808

[18] A. V. Dorn , R. E. Cooney , M. L. Sabin , Lancet 2020, 395, 1243. 32305087 10.1016/S0140-6736(20)30893-X PMC7162639

[19] Y.‐W. Tang , J. E. Schmitz , D. H. Persing , C. W. Stratton , J. Clin. Microbiol. 2020, 58, e00512. 32245835 10.1128/JCM.00512-20 PMC7269383

[20] I. F. Miller , A. D. Becker , B. T. Grenfell , C. J. E. Metcalf , Nat. Med. 2020, 26, 1212. 32546823 10.1038/s41591-020-0952-y

[21] W. E. Allen , H. Altae‐Tran , J. Briggs , X. Jin , G. McGee , A. Shi , R. Raghavan , M. Kamariza , N. Nova , A. Pereta , C. Danford , A. Kamel , P. Gothe , E. Milam , J. Aurambault , T. Primke , W. Li , J. Inkenbrandt , T. Huynh , E. Chen , C. Lee , M. Croatto , H. Bentley , W. Lu , R. Murray , M. Travassos , B. A. Coull , J. Openshaw , C. S. Greene , O. Shalem , G. King , R. Probasco , D. R. Cheng , B. Silbermann , F. Zhang , X. Lin , Nat. Hum. Behav. 2020, 4, 972. 32848231 10.1038/s41562-020-00944-2 PMC7501153

[22] M. Whitaker , J. Elliott , M. Chadeau‐Hyam , S. Riley , A. Darzi , G. Cooke , H. Ward , P. Elliott , Nat. Commun. 2022, 13, 1957. 35413949 10.1038/s41467-022-29521-z PMC9005552

[23] C. Menni , C. H. Sudre , C. J. Steves , S. Ourselin , T. D. Spector , Lancet 2020, 395, e107. 32505221 10.1016/S0140-6736(20)31281-2 PMC7272184

[24] K. Lamote , E. Janssens , E. Schillebeeckx , T. S. Lapperre , B. Y. De Winter , J. P. van Meerbeeck , J. Breath Res. 2020, 14, 042001. 32599571 10.1088/1752-7163/aba105

[25] G. Giovannini , H. Haick , D. Garoli , ACS Sensors 2021, 6, 1408. 33825440 10.1021/acssensors.1c00312 PMC8043202

[26] H. Jeong , J. A. Rogers , S. Xu , Sci. Adv. 2020, 6, eabd4794. 32917604 10.1126/sciadv.abd4794 PMC7467694

[27] Q. Yang , T.‐L. Liu , Y. Xue , H. Wang , Y. Xu , B. Emon , M. Wu , C. Rountree , T. Wei , I. Kandela , C. R. Haney , A. Brikha , I. Stepien , J. Hornick , R. A. Sponenburg , C. Cheng , L. Ladehoff , Y. Chen , Z. Hu , C. Wu , M. Han , J. M. Torkelson , Y. Kozorovitskiy , M. T. A. Saif , Y. Huang , J.‐K. Chang , J. A. Rogers , Nat. Electron. 2022, 5, 526.

[28] J. T. Reeder , Z. Xie , Q. Yang , M.‐H. Seo , Y. Yan , Y. Deng , K. R. Jinkins , S. R. Krishnan , C. Liu , S. McKay , E. Patnaude , A. Johnson , Z. Zhao , M. J. Kim , Y. Xu , I. Huang , R. Avila , C. Felicelli , E. Ray , X. Guo , W. Z. Ray , Y. Huang , M. R. MacEwan , J. A. Rogers , Science 2022, 377, 109. 35771907 10.1126/science.abl8532

[29] Y. H. Jung , J.‐Y. Yoo , A. Vázquez‐Guardado , J.‐H. Kim , J.‐T. Kim , H. Luan , M. Park , J. Lim , H.‐S. Shin , C.‐J. Su , R. Schloen , J. Trueb , R. Avila , J.‐K. Chang , D. S. Yang , Y. Park , H. Ryu , H.‐J. Yoon , G. Lee , H. Jeong , J. U. Kim , A. Akhtar , J. Cornman , T.‐i. Kim , Y. Huang , J. A. Rogers , Nat. Electron. 2022, 5, 374.

[30] H. Yuk , C. E. Varela , C. S. Nabzdyk , X. Mao , R. F. Padera , E. T. Roche , X. Zhao , Nature 2019, 575, 169. 31666696 10.1038/s41586-019-1710-5

[31] J. Deng , H. Yuk , J. Wu , C. E. Varela , X. Chen , E. T. Roche , C. F. Guo , X. Zhao , Nat. Mater. 2021, 20, 229. 32989277 10.1038/s41563-020-00814-2

[32] L. Jiang , G. Lu , Y. Zeng , Y. Sun , H. Kang , J. Burford , C. Gong , M. S. Humayun , Y. Chen , Q. Zhou , Nat. Commun. 2022, 13, 3853. 35788594 10.1038/s41467-022-31599-4 PMC9253314

[33] W. Li , T. Yang , C. Liu , Y. Huang , C. Chen , H. Pan , G. Xie , H. Tai , Y. Jiang , Y. Wu , Z. Kang , L.‐Q. Chen , Y. Su , Z. Hong , Adv. Sci. 2022, 9, 2105550. 10.1002/advs.202105550 PMC9069389 35277947

[34] Y. Wang , H. Haick , S. Guo , C. Wang , S. Lee , T. Yokota , T. Someya , Chem. Soc. Rev. 2022, 51, 3759. 35420617 10.1039/d2cs00207h

[35] Y. Su , J. Wang , B. Wang , T. Yang , B. Yang , G. Xie , Y. Zhou , S. Zhang , H. Tai , Z. Cai , G. Chen , Y. Jiang , L.‐Q. Chen , J. Chen , ACS Nano 2020, 14, 6067. 32271532 10.1021/acsnano.0c01804

[36] Y. Su , C. Chen , H. Pan , Y. Yang , G. Chen , X. Zhao , W. Li , Q. Gong , G. Xie , Y. Zhou , S. Zhang , H. Tai , Y. Jiang , J. Chen , Adv. Funct. Mater. 2021, 31, 2010962.

[37] G. Chen , C. Au , J. Chen , Trends Biotechnol. 2021, 39, 1078. 33551177 10.1016/j.tibtech.2020.12.011

[38] G. Chen , Y. Fang , X. Zhao , T. Tat , J. Chen , Nat. Electron. 2021, 4, 175.

[39] A. Libanori , G. Chen , X. Zhao , Y. Zhou , J. Chen , Nat. Electron. 2022, 5, 142. 10.1038/s41928-022-00723-z PMC13309183 42367254

[40] K. Meng , X. Xiao , W. Wei , G. Chen , A. Nashalian , S. Shen , X. Xiao , J. Chen , Adv. Mater. 2022, 34, 2109357. 10.1002/adma.202109357 35044014

[41] Z. Zhou , K. Chen , X. Li , S. Zhang , Y. Wu , Y. Zhou , K. Meng , C. Sun , Q. He , W. Fan , E. Fan , Z. Lin , X. Tan , W. Deng , J. Yang , J. Chen , Nat. Electron. 2020, 3, 571.

[42] K. Meng , S. Zhao , Y. Zhou , Y. Wu , S. Zhang , Q. He , X. Wang , Z. Zhou , W. Fan , X. Tan , J. Yang , J. Chen , Matter 2020, 2, 896.

[43] W. Yan , G. Noel , G. Loke , E. Meiklejohn , T. Khudiyev , J. Marion , G. Rui , J. Lin , J. Cherston , A. Sahasrabudhe , J. Wilbert , I. Wicaksono , R. W. Hoyt , A. Missakian , L. Zhu , C. Ma , J. Joannopoulos , Y. Fink , Nature 2022, 603, 616. 35296860 10.1038/s41586-022-04476-9

[44] Y. Su , W. Li , X. Cheng , Y. Zhou , S. Yang , X. Zhang , C. Chen , T. Yang , H. Pan , G. Xie , G. Chen , X. Zhao , X. Xiao , B. Li , H. Tai , Y. Jiang , L.‐Q. Chen , F. Li , J. Chen , Nat. Commun. 2022, 13, 4867. 35982033 10.1038/s41467-022-32518-3 PMC9388583

[45] S. Zhang , M. Bick , X. Xiao , G. Chen , A. Nashalian , J. Chen , Matter 2021, 4, 845.

[46] G. Conta , A. Libanori , T. Tat , G. Chen , J. Chen , Adv. Mater. 2021, 33, 2007502. 10.1002/adma.202007502 34014583

[47] Y. S. Choi , H. Jeong , R. T. Yin , R. Avila , A. Pfenniger , J. Yoo , J. Y. Lee , A. Tzavelis , Y. J. Lee , S. W. Chen , H. S. Knight , S. Kim , H.‐Y. Ahn , G. Wickerson , A. Vázquez‐Guardado , E. Higbee‐Dempsey , B. A. Russo , M. A. Napolitano , T. J. Holleran , L. A. Razzak , A. N. Miniovich , G. Lee , B. Geist , B. Kim , S. Han , J. A. Brennan , K. Aras , S. S. Kwak , J. Kim , E. A. Waters , X. Yang , A. Burrell , K. San Chun , C. Liu , C. Wu , A. Y. Rwei , A. N. Spann , A. Banks , D. Johnson , Z. J. Zhang , C. R. Haney , S. H. Jin , A. V. Sahakian , Y. Huang , G. D. Trachiotis , B. P. Knight , R. K. Arora , I. R. Efimov , J. A. Rogers , Science 2022, 376, 1006. 35617386 10.1126/science.abm1703 PMC9282941

[48] M. Berggren , E. D. Głowacki , D. T. Simon , E. Stavrinidou , K. Tybrandt , Chem. Rev. 2022, 122, 4826. 35050623 10.1021/acs.chemrev.1c00390 PMC8874920

[49] R. Avila , C. Li , Y. Xue , J. A. Rogers , Y. Huang , Proc. Natl. Acad. Sci. U.S.A. 2021, 118, e2026405118. 33836613 10.1073/pnas.2026405118 PMC7980470

[50] H. Joo , Y. Lee , J. Kim , J.‐S. Yoo , S. Yoo , S. Kim , A. K. Arya , S. Kim , S. H. Choi , N. Lu , H. S. Lee , S. Kim , S.‐T. Lee , D.‐H. Kim , Sci. Adv. 2021, 7, eabd4639. 33523849 10.1126/sciadv.abd4639 PMC7775752

[51] M. Amjadi , S. Sheykhansari , B. J. Nelson , M. Sitti , Adv. Mater. 2018, 30, 1704530. 10.1002/adma.201704530 29315905

[52] G. Chen , Y. Li , M. Bick , J. Chen , Chem. Rev. 2020, 120, 3668. 32202762 10.1021/acs.chemrev.9b00821

[53] N. Zhang , F. Huang , S. Zhao , X. Lv , Y. Zhou , S. Xiang , S. Xu , Y. Li , G. Chen , C. Tao , Y. Nie , J. Chen , X. Fan , Matter 2020, 2, 1260.

[54] W. Deng , Y. Zhou , A. Libanori , G. Chen , W. Yang , J. Chen , Chem. Soc. Rev. 2022, 51, 3380. 35352069 10.1039/d1cs00858g

[55] J. He , C. Lu , H. Jiang , F. Han , X. Shi , J. Wu , L. Wang , T. Chen , J. Wang , Y. Zhang , H. Yang , G. Zhang , X. Sun , B. Wang , P. Chen , Y. Wang , Y. Xia , H. Peng , Nature 2021, 597, 57. 34471277 10.1038/s41586-021-03772-0

[56] M. Liao , C. Wang , Y. Hong , Y. Zhang , X. Cheng , H. Sun , X. Huang , L. Ye , J. Wu , X. Shi , X. Kang , X. Zhou , J. Wang , P. Li , X. Sun , P. Chen , B. Wang , Y. Wang , Y. Xia , Y. Cheng , H. Peng , Nat. Nanotechnol. 2022, 17, 372. 35058651 10.1038/s41565-021-01062-4

[57] C. Chen , J. Feng , J. Li , Y. Guo , X. Shi , H. Peng , Chem. Rev. 2022, DOI: 10.1021/acs.chemrev.2c00192. 35977344

[58] Y. Fang , G. Chen , M. Bick , J. Chen , Chem. Soc. Rev. 2021, 50, 9357. 34296235 10.1039/d1cs00003a

[59] C. Wang , X. Chen , L. Wang , M. Makihata , H.‐C. Liu , T. Zhou , X. Zhao , Science 2022, 377, 517. 35901155 10.1126/science.abo2542

[60] T. Dinh , T. Nguyen , H.‐P. Phan , N.‐T. Nguyen , D. V. Dao , J. Bell , Biosens. Bioelectron. 2020, 166, 112460. 32862846 10.1016/j.bios.2020.112460

[61] C. Chen , M. Jiang , X. Luo , H. Tai , Y. Jiang , M. Yang , G. Xie , Y. Su , Sens. Actuators B Chem. 2022, 370, 132441.

[62] S. Chen , G. Qian , B. Ghanem , Y. Wang , Z. Shu , X. Zhao , L. Yang , X. Liao , Y. Zheng , Adv. Sci., 2022, 9, 2203460. 10.1002/advs.202203460 PMC9661834 36089657

[63] G. Chen , X. Xiao , X. Zhao , T. Tat , M. Bick , J. Chen , Chem. Rev. 2022, 122, 3259. 34939791 10.1021/acs.chemrev.1c00502

[64] G. Quer , J. M. Radin , M. Gadaleta , K. Baca‐Motes , L. Ariniello , E. Ramos , V. Kheterpal , E. J. Topol , S. R. Steinhubl , Nat. Med. 2021, 27, 73. 33122860 10.1038/s41591-020-1123-x

[65] J. Daniels , S. Wadekar , K. DeCubellis , G. W. Jackson , A. S. Chiu , Q. Pagneux , H. Saada , I. Engelmann , J. Ogiez , D. Loze‐Warot , R. Boukherroub , S. Szunerits , Biosens. Bioelectron. 2021, 192, 113486. 34260968 10.1016/j.bios.2021.113486 PMC8264268

[66] X. Ni , W. Ouyang , H. Jeong , J.‐T. Kim , A. Tzavelis , A. Mirzazadeh , C. Wu , J. Y. Lee , M. Keller , C. K. Mummidisetty , M. Patel , N. Shawen , J. Huang , H. Chen , S. Ravi , J.‐K. Chang , K. Lee , Y. Wu , F. Lie , Y. J. Kang , J. U. Kim , L. P. Chamorro , A. R. Banks , A. Bharat , A. Jayaraman , S. Xu , J. A. Rogers , Proc. Natl. Acad. Sci. U.S.A. 2021, 118, e2026610118. 33893178 10.1073/pnas.2026610118 PMC8126790

[67] B. Shan , Y. Y. Broza , W. Li , Y. Wang , S. Wu , Z. Liu , J. Wang , S. Gui , L. Wang , Z. Zhang , W. Liu , S. Zhou , W. Jin , Q. Zhang , D. Hu , L. Lin , Q. Zhang , W. Li , J. Wang , H. Liu , Y. Pan , H. Haick , ACS Nano 2020, 14, 12125. 32808759 10.1021/acsnano.0c05657

[68] P. Aveyard , M. Gao , N. Lindson , J. Hartmann‐Boyce , P. Watkinson , D. Young , C. A. C. Coupland , P. S. Tan , A. K. Clift , D. Harrison , D. W. Gould , I. D. Pavord , J. Hippisley‐Cox , Lancet Respir. Med. 2021, 9, 909. 33812494 10.1016/S2213-2600(21)00095-3 PMC8016404

[69] B. Long , W. J. Brady , A. Koyfman , M. Gottlieb , Am. J. Emerg. Med. 2020, 38, 1504. 32317203 10.1016/j.ajem.2020.04.048 PMC7165109

[70] E. Fraser , BMJ 2020, 370, m3001. 32747332 10.1136/bmj.m3001

[71] H. Jeong , J. Y. Lee , K. Lee , Y. J. Kang , J.‐T. Kim , R. Avila , A. Tzavelis , J. Kim , H. Ryu , S. S. Kwak , J. U. Kim , A. Banks , H. Jang , J.‐K. Chang , S. Li , C. K. Mummidisetty , Y. Park , S. Nappi , K. S. Chun , Y. J. Lee , K. Kwon , X. Ni , H. U. Chung , H. Luan , J.‐H. Kim , C. Wu , S. Xu , A. Banks , A. Jayaraman , Y. Huang , J. A. Rogers , Sci. Adv. 2021, 7, eabg3092. 33980495 10.1126/sciadv.abg3092 PMC8115927

[72] Z. Zhao , C. Yan , Z. Liu , X. Fu , L.‐M. Peng , Y. Hu , Z. Zheng , Adv. Mater. 2016, 28, 10267. 27690188 10.1002/adma.201603679

[73] Y. Fang , J. Xu , X. Xiao , Y. Zou , X. Zhao , Y. Zhou , J. Chen , Adv. Mater. 2022, 34, 2200252. 10.1002/adma.202200252 35306703

[74] M. Wang , J. Zhang , Y. Tang , J. Li , B. Zhang , E. Liang , Y. Mao , X. Wang , ACS Nano 2018, 12, 6156. 29847095 10.1021/acsnano.8b02562 PMC6279609

[75] X. Chen , X. Li , J. Shao , N. An , H. Tian , C. Wang , T. Han , L. Wang , B. Lu , Small 2017, 13, 1604245. 10.1002/smll.201604245 28452402

[76] Y. Su , W. Li , L. Yuan , C. Chen , H. Pan , G. Xie , G. Conta , S. Ferrier , X. Zhao , G. Chen , H. Tai , Y. Jiang , J. Chen , Nano Energy 2021, 89, 106321.

[77] Y. Zhou , X. Zhao , J. Xu , Y. Fang , G. Chen , Y. Song , S. Li , J. Chen , Nat. Mater. 2021, 20, 1301. 10.1038/s41563-021-01093-1 34594013

[78] X. Zhao , Y. Zhou , J. Xu , G. Chen , Y. Fang , T. Tat , J. Chen , Nat. Commun. 2021, 12, 6839. 34799591 10.1038/s41467-021-27066-1 PMC8604991

[79] G. Chen , X. Zhao , S. Andalib , J. Xu , Y. Zhou , T. Tat , K. Lin , J. Chen , Matter 2021, 4, 3725. 35846392 10.1016/j.matt.2021.09.012 PMC9281417

[80] L. Chen , M. Lu , H. Yang , J. R. Salas Avila , B. Shi , L. Ren , G. Wei , X. Liu , W. Yin , ACS Nano 2020, 14, 8191. 32520522 10.1021/acsnano.0c01643

[81] Y.‐S. Kim , J. Lu , B. Shih , A. Gharibans , Z. Zou , K. Matsuno , R. Aguilera , Y. Han , A. Meek , J. Xiao , M. T. Tolley , T. P. Coleman , Adv. Mater. 2017, 29, 1701312. 10.1002/adma.201701312 28837756

[82] A. Hermawan , T. Amrillah , A. Riapanitra , W.‐J. Ong , S. Yin , Adv. Healthcare Mater. 2021, 10, 2100970. 10.1002/adhm.202100970 34318999

[83] J. Dai , L. Li , B. Shi , Z. Li , Biosens. Bioelectron. 2021, 194, 113609. 34509719 10.1016/j.bios.2021.113609

[84] Y. Su , G. Chen , C. Chen , Q. Gong , G. Xie , M. Yao , H. Tai , Y. Jiang , J. Chen , Adv. Mater. 2021, 33, 2101262. 10.1002/adma.202101262 34240473

[85] X. Zhao , G. Chen , Y. Zhou , A. Nashalian , J. Xu , T. Tat , Y. Song , A. Libanori , S. Xu , S. Li , J. Chen , ACS Nano 2022, 16, 6013. 35417654 10.1021/acsnano.1c11350

[86] X. Zhao , A. Nashalian , I. W. Ock , S. Popoli , J. Xu , J. Yin , T. Tat , A. Libanori , G. Chen , Y. Zhou , J. Chen , Adv. Mater. 2022, 34, 2204238. 10.1002/adma.202204238 35918815

[87] J. Xu , T. Tat , X. Zhao , Y. Zhou , D. Ngo , X. Xiao , J. Chen , Appl. Phys. Rev. 2022, 9, 031404.

[88] Q. Xu , Y. Fang , Q. Jing , N. Hu , K. Lin , Y. Pan , L. Xu , H. Gao , M. Yuan , L. Chu , Y. Ma , Y. Xie , J. Chen , L. Wang , Biosens. Bioelectron. 2021, 187, 113329. 34020223 10.1016/j.bios.2021.113329 PMC8118703

[89] A. S. Ginsburg , J. L. Lenahan , R. Izadnegahdar , J. M. Ansermino , Am. J. Respir. Crit. Care Med. 2018, 197, 1116. 29474107 10.1164/rccm.201711-2233CI

[90] S. Grassin‐Delyle , C. Roquencourt , P. Moine , G. Saffroy , S. Carn , N. Heming , J. Fleuriet , H. Salvator , E. Naline , L.‐J. Couderc , P. Devillier , E. A. Thévenot , D. Annane , EBioMedicine 2021, 63, 103154. 33279860 10.1016/j.ebiom.2020.103154 PMC7714658

[91] O. Gould , N. Ratcliffe , E. Król , B. de Lacy Costello , J. Breath Res. 2020, 14, 041001. 32531777 10.1088/1752-7163/ab9c32

[92] T. R. Ray , J. Choi , A. J. Bandodkar , S. Krishnan , P. Gutruf , L. Tian , R. Ghaffari , J. A. Rogers , Chem. Rev. 2019, 119, 5461. 30689360 10.1021/acs.chemrev.8b00573

[93] Y. Yu , J. Li , S. A. Solomon , J. Min , J. Tu , W. Guo , C. Xu , Y. Song , W. Gao , Sci. Robot. 2022, 7, eabn0495. 35648844 10.1126/scirobotics.abn0495 PMC9302713

[94] Y. Yang , W. Gao , Chem. Soc. Rev. 2019, 48, 1465. 29611861 10.1039/c7cs00730b

[95] S. M. Moghadas , M. C. Fitzpatrick , P. Sah , A. Pandey , A. Shoukat , B. H. Singer , A. P. Galvani , Proc. Natl. Acad. Sci. U.S.A. 2020, 117, 17513. 32632012 10.1073/pnas.2008373117 PMC7395516

[96] W. Ibrahim , R. L. Cordell , M. J. Wilde , M. Richardson , L. Carr , A. Sundari Devi Dasi , B. Hargadon , R. C. Free , P. S. Monks , C. E. Brightling , N. J. Greening , S. Siddiqui , ERJ Open Res. 2021, 7, 00139. 34235208 10.1183/23120541.00139-2021 PMC8255539

[97] M. Sawano , K. Takeshita , H. Ohno , H. Oka , J. Breath Res. 2021, 15, 037103. 10.1088/1752-7163/ac0414 34020435

[98] H. Chen , X. Qi , L. Zhang , X. Li , J. Ma , C. Zhang , H. Feng , M. Yao , J. Breath Res. 2021, 15, 047104. 10.1088/1752-7163/ac2e57 34624875

[99] V. V. Tran , N. H. T. Tran , H. S. Hwang , M. Chang , Biosens. Bioelectron. 2021, 182, 113192. 33819902 10.1016/j.bios.2021.113192 PMC7992312

[100] Q. Xue , X. Kan , Z. Pan , Z. Li , W. Pan , F. Zhou , X. Duan , Biosens. Bioelectron. 2021, 186, 113286. 33990035 10.1016/j.bios.2021.113286 PMC8091738

[101] S. D. Bukkitgar , N. P. Shetti , T. M. Aminabhavi , Chem. Eng. J. 2021, 420, 127575. 33162783 10.1016/j.cej.2020.127575 PMC7605744

[102] G. Seo , G. Lee , M. J. Kim , S.‐H. Baek , M. Choi , K. B. Ku , C.‐S. Lee , S. Jun , D. Park , H. G. Kim , S.‐J. Kim , J.‐O. Lee , B. T. Kim , E. C. Park , S. I. Kim , ACS Nano 2020, 14, 5135. 32293168 10.1021/acsnano.0c02823

[103] B. Liu , A. Libanori , Y. Zhou , X. Xiao , G. Xie , X. Zhao , Y. Su , S. Wang , Z. Yuan , Z. Duan , J. Liang , Y. Jiang , H. Tai , J. Chen , ACS Appl. Mater. Interfaces. 2022, 14, 7301. 35076218 10.1021/acsami.1c22457

